# Age-Specific Efficacy of Platelet-Rich Plasma in Tonsillectomy: A Systematic Review and Meta-Analysis

**DOI:** 10.3390/jcm15166182

**Published:** 2026-08-10

**Authors:** Ji-Sun Kim, Gulnaz Stybayeva, Se Hwan Hwang

**Affiliations:** 1 Department of Otolaryngology-Head and Neck Surgery, Eunpyeong St. Mary’s Hospital, College of Medicine, The Catholic University of Korea, Seoul 03312, Republic of Korea; skswltjs23@catholic.ac.kr; 2 Department of Physiology and Biomedical Engineering, Mayo Clinic, Rochester, MN 55905, USA; stybayeva.gulnaz@mayo.edu; 3 Department of Otolaryngology-Head and Neck Surgery, Bucheon St. Mary’s Hospital, College of Medicine, The Catholic University of Korea, Bucheon 14647, Republic of Korea

**Keywords:** platelet-rich plasma, tonsillectomy, pain, postoperative, wound healing, postoperative hemorrhage, meta-analysis

## Abstract

**Background/Objectives**: The objective of this study was to evaluate age-specific effects of intraoperative platelet-rich plasma (PRP) administration on postoperative pain, wound healing, and bleeding after tonsillectomy. **Methods**: PubMed, Embase, Scopus, Google Scholar, and Cochrane databases were searched from inception to October 2025. Randomized controlled trials comparing intraoperative PRP with placebo or no treatment in patients undergoing tonsillectomy were included. Data on postoperative pain, wound healing, and bleeding were extracted and analyzed using standardized mean differences (SMDs) and odds ratios (ORs) with 95% confidence intervals (CIs). Subgroup analyses were performed by age (adults versus children) and by postoperative day. **Results**: Nine randomized controlled trials comprising 442 participants were included. In adults, PRP significantly reduced postoperative pain (SMD = −1.26 [−1.70 to −0.81]) and improved wound healing (SMD = −1.43 [−2.65 to −0.22]). The analgesic effect was greatest during the early recovery period (postoperative days 0–5) and diminished thereafter. There were no significant differences in primary postoperative bleeding (OR = 0.49 [0.04 to 5.61]) or secondary postoperative bleeding (OR = 0.23 [0.02 to 2.16]) between the PRP and control groups. In children, PRP did not significantly affect pain or bleeding outcomes. **Conclusions**: Intraoperative PRP may reduce early postoperative pain and may promote mucosal recovery in adults. However, its clinical efficacy in children remains unclear. Further well-designed, age-stratified trials are warranted to clarify the role of PRP in enhancing postoperative recovery.

## 1. Introduction

Tonsillectomy remains one of the most frequently performed surgical procedures in otolaryngology, primarily indicated for recurrent throat infections and obstructive sleep-disordered breathing [1]. Although tonsillectomy offers well-established therapeutic benefits, it is accompanied by postoperative morbidities such as throat pain, nausea, vomiting, and bleeding, which require careful perioperative management and patient counseling [2,3]. The updated clinical practice guideline from the American Academy of Otolaryngology–Head and Neck Surgery Foundation identifies postoperative pain and hemorrhage as the major complications of tonsillectomy and recommends intraoperative dexamethasone and postoperative acetaminophen or ibuprofen for optimal pain control [4]. Reported rates of primary bleeding (within 24 h after surgery) range from 0.2% to 2.2%, whereas secondary bleeding (beyond 24 h) occurs in 0.1% to 3% of patients, occasionally necessitating hospital readmission or surgical intervention [5]. Despite these standardized approaches, postoperative pain and bleeding continue to affect recovery and patient quality of life. Therefore, adjunctive treatments that enhance mucosal healing and alleviate discomfort may improve postoperative outcomes.

Platelet-rich plasma (PRP) is an autologous blood-derived product enriched with platelets that release numerous growth factors involved in hemostasis and tissue repair [6]. These bioactive molecules, including platelet-derived and vascular endothelial growth factors, stimulate cell proliferation, angiogenesis, and epithelial regeneration [6,7]. PRP also modulates inflammation through cytokine regulation and enhances early wound healing by promoting macrophage activity and vascular permeability [6]. Since its introduction in the late 1990s, PRP has been increasingly applied across multiple surgical specialties, including oral and maxillofacial, orthopedic, plastic, and otolaryngologic surgery, as an adjunct to facilitate postoperative wound healing and reduce morbidity [6,8,9].

A recent meta-analysis reported that intraoperative PRP significantly reduced early postoperative pain and overall post-tonsillectomy hemorrhage [10]. However, that analysis combined adult and pediatric populations, despite clinically important differences in postoperative recovery between these groups. Differences have been reported in postoperative pain severity, recovery patterns, and postoperative complications between adult and pediatric patients [5,11]. Adults generally experience a more prolonged and painful recovery, whereas children often demonstrate more rapid restoration of oral intake and daily activity. Therefore, an intervention that improves postoperative recovery may not provide the same degree of benefit in adult and pediatric populations. In addition, recently published randomized controlled trials have provided further information regarding postoperative pain and wound healing. A more detailed evaluation stratified by adult and pediatric populations and by postoperative time point is therefore needed to determine whether the potential benefits of PRP are consistent across these patient groups and throughout the recovery period.

The present systematic review and meta-analysis aimed to evaluate the effects of intraoperative PRP on postoperative pain, wound healing, and bleeding after tonsillectomy. Adult and pediatric populations were analyzed separately, and pain and wound healing outcomes were further evaluated according to postoperative day. By incorporating recent randomized controlled trials and examining the temporal pattern of treatment effects, this study sought to provide a more clinically relevant assessment of the potential role of PRP in perioperative tonsillectomy management.

## 2. Materials and Methods

This systematic review and meta-analysis was conducted in accordance with the Preferred Reporting Items for Systematic Reviews and Meta-Analyses (PRISMA) 2020 guidelines (Supplementary Table S1). The study protocol was developed according to the Population, Intervention, Comparison, Outcomes, and Study design (PICOS) framework to ensure methodological transparency.

### 2.1. Search Strategy and Study Selection

The PICOS criteria for this review were defined as follows: (1) Population: patients who underwent tonsillectomy; (2) Intervention: intraoperative platelet-rich plasma (PRP) application; (3) Comparison: placebo or no treatment; (4) Outcomes: postoperative pain score, tonsillectomy bed healing state, and incidence of postoperative bleeding (primary or secondary); and (5) Study Design: randomized controlled trials. Electronic databases including PubMed, Embase, Scopus, Google Scholar, and the Cochrane Central Register of Controlled Trials were searched from inception to October 2025. The search strategy combined the terms “platelet-rich plasma,” “PRP,” “tonsillectomy,” “adenotonsillectomy,” “pain,” and “bleeding.” Searches were restricted to English-language articles. The complete search strategies for each database are provided in Supplementary Table S2.

The searches were conducted with the assistance of a medical librarian with over 10 years of experience. To ensure comprehensive coverage, the authors also manually reviewed the reference lists of all relevant publications to identify additional eligible studies. Two reviewers independently screened titles and abstracts, and full texts were retrieved when eligibility could not be determined from the abstract. Studies unrelated to PRP application during tonsillectomy were excluded. Discrepancies were resolved through discussion or, if necessary, by consultation with a third reviewer.

**Figure 1:**
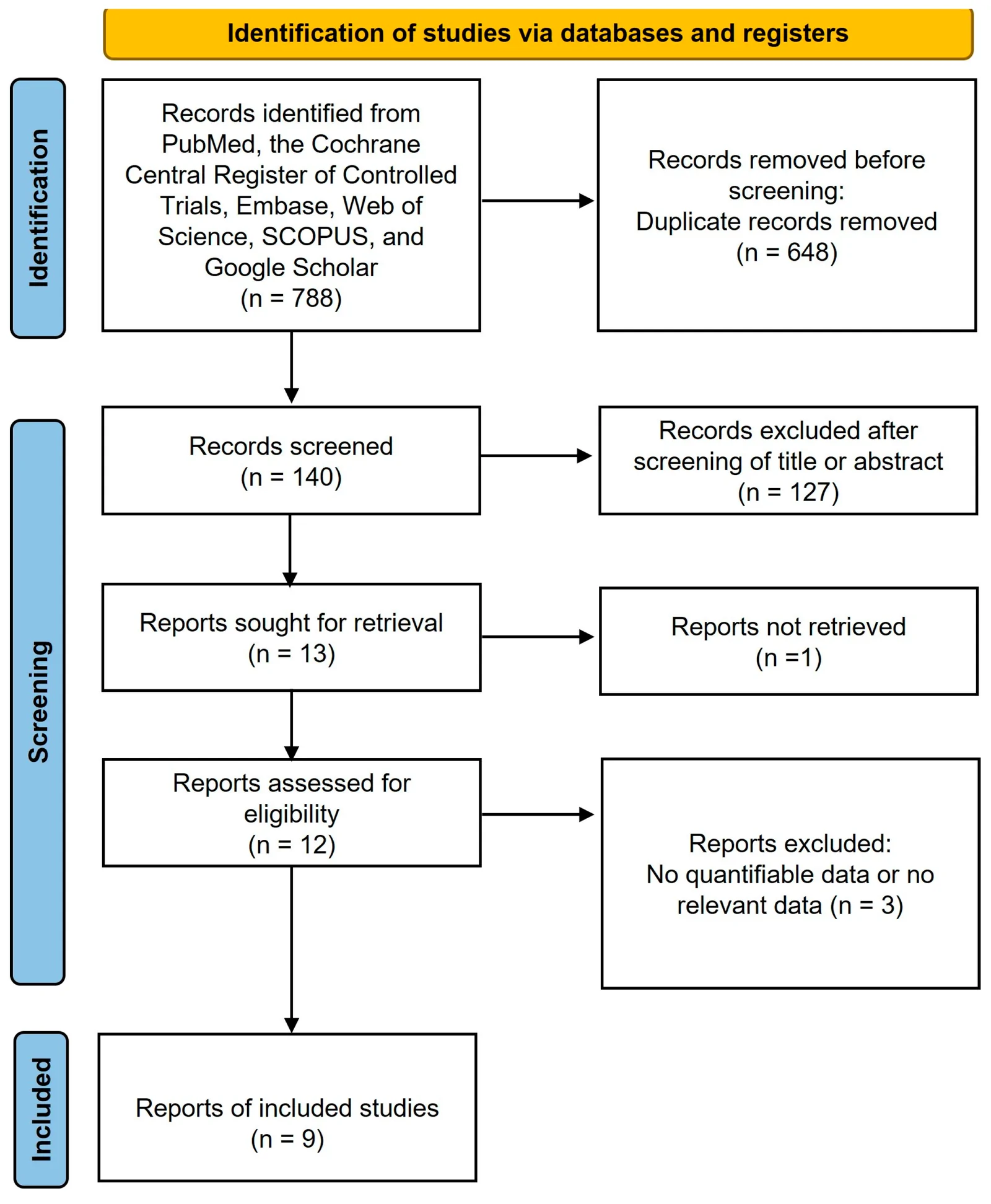
Study selection diagram.

Studies were included if they met the following criteria: (1) randomized controlled design; (2) tonsillectomy performed with or without adenoidectomy; (3) intraoperative or perioperative use of autologous PRP; (4) a placebo or no-treatment control; and (5) quantitative data for at least one prespecified outcome (pain, wound healing, or postoperative bleeding). Studies were excluded if PRP was used in combination with other adjunctive agents such as fibrin sealant, if additional surgical procedures such as uvulopalatopharyngoplasty were performed, or if quantitative outcome data were unavailable. The study selection process followed the PRISMA 2020 flow structure (Figure 1).

### 2.2. Data Extraction and Risk-of-Bias Assessment

Data extraction was conducted using standardized forms [12,13]. The sample size extracted from each study represented the number of participants included in the quantitative analyses. When outcome data were unavailable, those participants were excluded from the corresponding quantitative analyses. The primary outcomes analyzed were postoperative pain scores, wound-healing status of the tonsillar bed, and the incidence of postoperative bleeding, which was categorized as primary (within 24 h postoperatively) or secondary (after 24 h postoperatively). These outcomes were compared between the PRP-treated and control groups (placebo or no treatment) during the perioperative period. Wound healing was evaluated based on epithelialization and surface appearance of the tonsillar fossae, typically graded on a four- or five-point ordinal scale. Postoperative pain was assessed using the Visual Analogue Scale (VAS) for adults and children aged seven years or older (0 = no pain, 10 = unbearable pain), and the Faces Pain Scale for children younger than seven years [14]. For each eligible study, data were extracted on the number of participants, age range, tonsillectomy technique, PRP preparation and administration method, volume of autologous blood collected, duration of postoperative follow-up, comparator group, and outcome measures. The extracted information was used to assess the methodological consistency of the included trials and to perform quantitative analyses of postoperative pain, wound-healing status, and incidence of postoperative bleeding.

The methodological quality of the included studies was assessed using the original Cochrane Risk of Bias tool (RoB 1.0), which assesses the adequacy of random sequence generation, allocation concealment, blinding, completeness of outcome data, and selective outcome reporting. Each domain was rated as low, unclear, or high risk of bias, and discrepancies between reviewers were resolved through discussion to reach consensus (Supplementary Table S3).

### 2.3. Statistical Analysis

All statistical analyses were performed using R software (version 4.3.1; R Foundation for Statistical Computing, Vienna, Austria). For continuous variables such as postoperative pain scores and wound healing grades, the effect size was expressed as the standardized mean difference (SMD) with corresponding 95% confidence intervals (CIs) to account for differences in outcome scales across studies. For dichotomous outcomes such as the incidence of postoperative bleeding, odds ratios (ORs) with 95% CIs were calculated. Several included trials used a split-mouth design, in which PRP was applied to one tonsillar bed and the comparator to the contralateral bed within the same patient. For these trials, each side was treated as an independent group in the pooled analyses.

Between-study heterogeneity was quantified using the I^2^ statistic, representing the percentage of total variation due to heterogeneity rather than chance. An I^2^ value above 50% was interpreted as substantial heterogeneity. When substantial heterogeneity was present (I^2^ > 50%), a random-effects model using the DerSimonian–Laird method was applied, whereas a fixed-effects model based on the inverse variance approach was used when heterogeneity was low (I^2^ ≤ 50%).

Exploratory assessment of funnel plot asymmetry using visual inspection and Egger’s regression test was performed. Because fewer than ten independent studies were included, the results were interpreted cautiously owing to the limited statistical power [15]. Leave-one-out sensitivity analyses were performed to evaluate the influence of individual studies on the pooled estimates.

## 3. Results

A total of nine randomized controlled trials comprising 442 participants were included in this meta-analysis [16,17,18,19,20,21,22,23,24]. The characteristics of the included studies are summarized in Table 1. The participant numbers presented in Table 1 correspond to those included in the quantitative analyses.

### 3.1. Effect of PRP on Postoperative Outcomes in Adults

**Figure 2:**
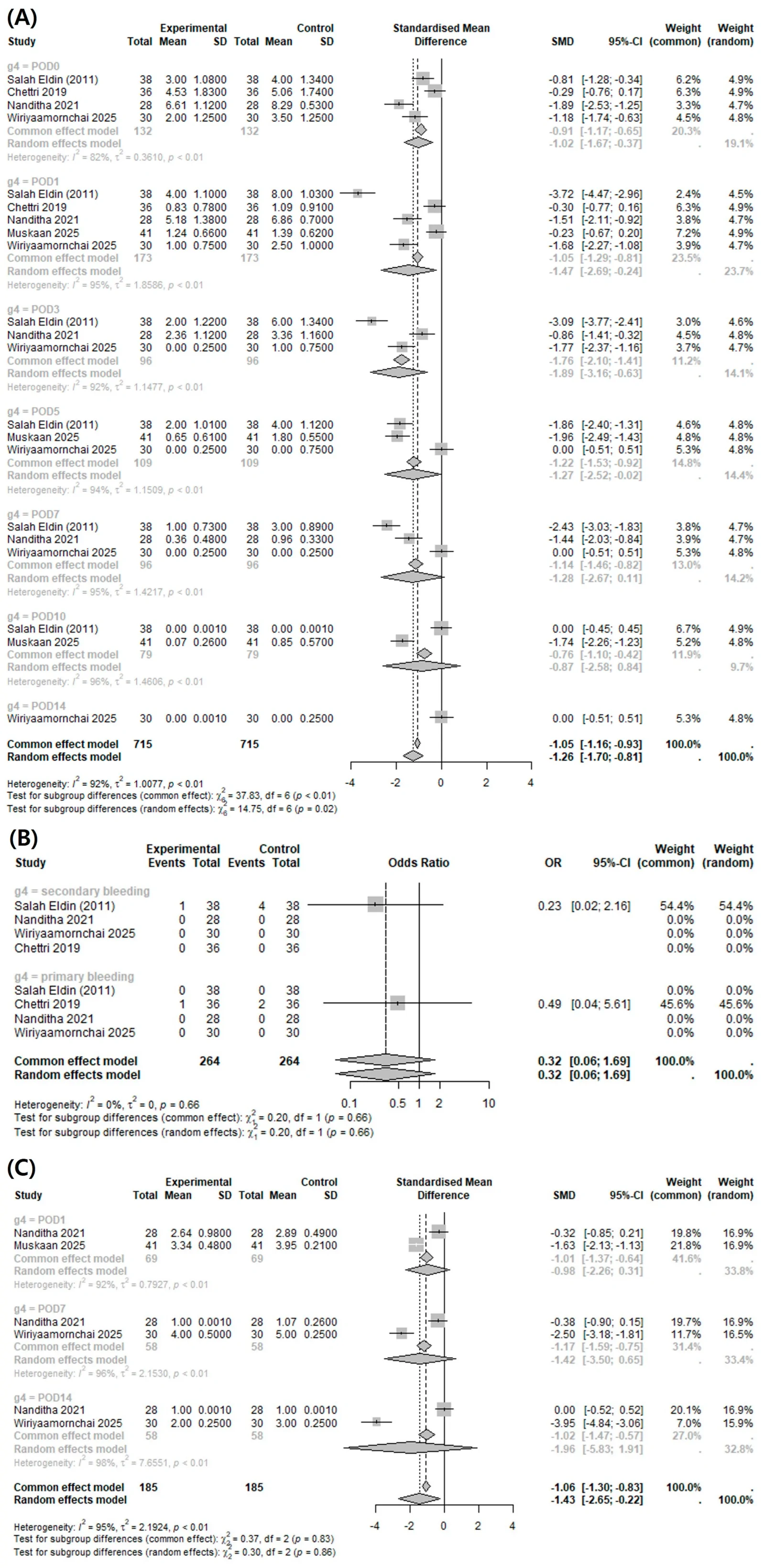
Forest plots of intraoperative platelet-rich plasma administration versus placebo in adults [17,19,20,23,24]. (**A**) SMDs of postoperative pain scores by postoperative day. (**B**) SMDs for tonsillar bed wound healing scores. (**C**) ORs comparing the incidence of primary (<24 h) and secondary (>24 h) postoperative bleeding. Abbreviations: SMD, standardized mean difference; OR, odds ratio; CI, confidence interval; POD, postoperative day.

Compared with the control group, intraoperative PRP administration significantly reduced postoperative pain and significantly improved wound healing, while no significant differences were observed in postoperative bleeding (Figure 2). Although substantial heterogeneity (I^2^ > 50%) was noted, these findings suggest a potential short-term symptomatic benefit of PRP, particularly during the early recovery phase.

In the pooled analysis, PRP application resulted in significantly lower postoperative pain scores than those of the control group (SMD = −1.26 [−1.70 to −0.81]; I^2^ = 92.2) (Figure 2A). Subgroup analyses by postoperative day (POD) demonstrated that the analgesic effect was most pronounced during the early recovery period. Pain reduction was statistically significant on POD 0 (SMD = −1.02 [−1.67 to −0.37]), POD 1 (SMD = −1.47 [−2.69 to −0.24]), POD 3 (SMD = −1.89 [−3.16 to −0.63]), and POD 5 (SMD = −1.27 [−2.52 to −0.02]). However, this effect diminished thereafter and was no longer significant at later time points, including POD 7 (SMD = −1.28 [−2.67 to 0.11]), POD 10 (SMD = −0.87 [−2.58 to 0.84]), and POD 14 (SMD = 0.00 [−0.51 to 0.51]) (Table 2).

PRP also significantly enhanced wound healing (SMD = −1.43 [−2.65 to −0.22]; I^2^ = 94.6) (Figure 2B). Although statistical significance was not reached at individual postoperative days, the direction of the effect indicated faster epithelialization in the PRP group, with results of POD 1 (SMD = −0.98 [−2.26 to 0.31]), POD 7 (SMD = −1.42 [−3.50 to 0.65]), and POD 14 (SMD = −1.96 [−5.83 to 1.91]) (Table 2).

There were no significant differences in the incidences of primary postoperative bleeding (OR = 0.49 [0.04 to 5.61]) or secondary postoperative bleeding (OR = 0.23 [0.02 to 2.16]) between the PRP and control groups (Figure 2C).

### 3.2. Effect of PRP on Postoperative Outcomes in Children

**Figure 3:**
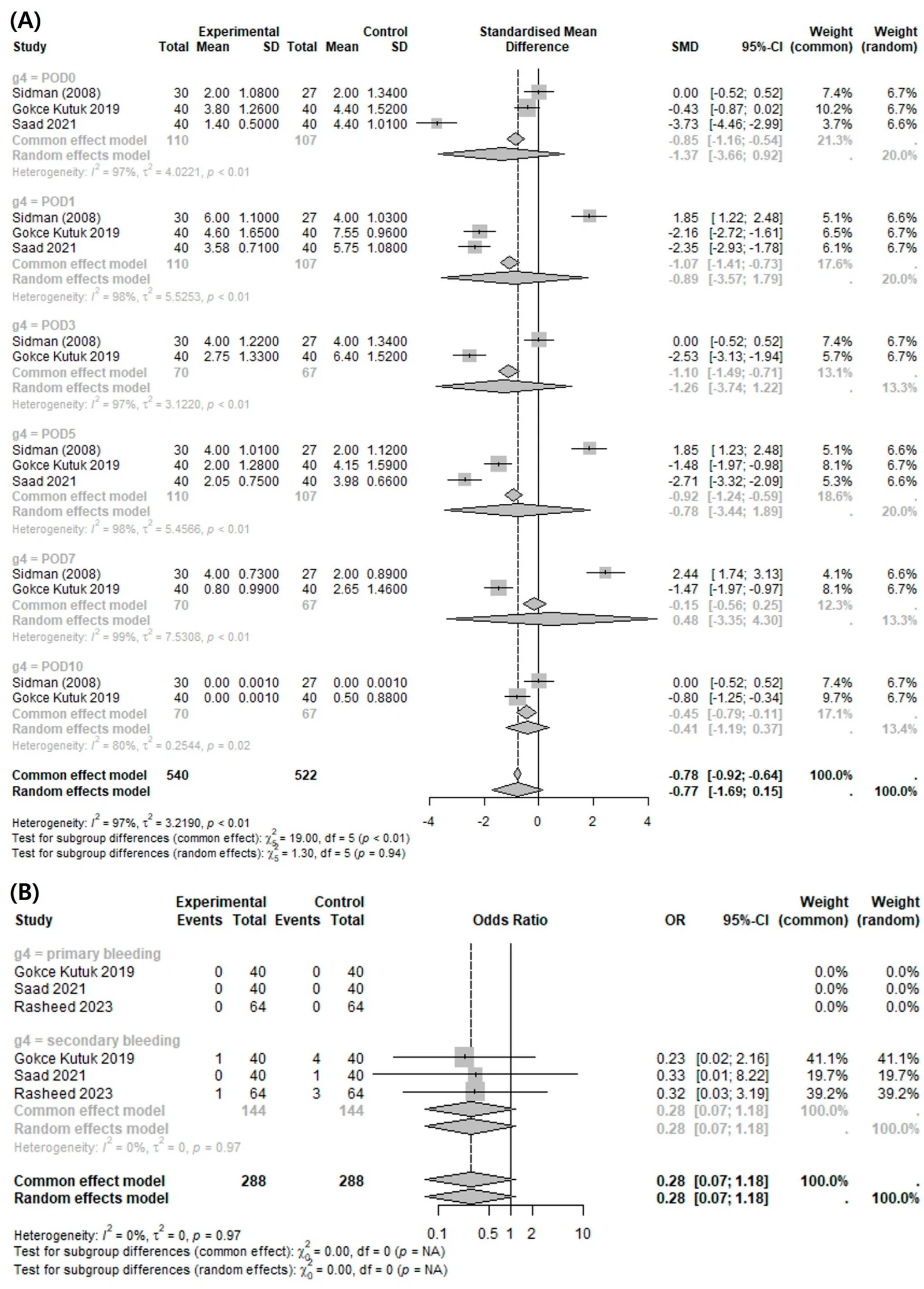
Forest plots of intraoperative platelet-rich plasma administration versus placebo in children [16,18,21,22]. (**A**) SMDs of postoperative pain scores by postoperative day. (**B**) ORs comparing the incidence of postoperative bleeding. Abbreviations: SMD, standardized mean difference; OR, odds ratio; CI, confidence interval; POD, postoperative day.

In the pediatric subgroup, intraoperative PRP administration did not significantly affect postoperative pain or bleeding compared with the control group (Figure 3).

The pooled analysis demonstrated no significant reduction in postoperative pain following PRP application compared with the control group (SMD = −0.77 [−1.69 to 0.15]; I^2^ = 96.9) (Figure 3A). Subgroup analyses by POD also showed no significant differences between the two groups, with results of POD 0 (SMD = −1.37 [−3.66 to 0.92]), POD 1 (SMD = −0.89 [−3.57 to 1.79]), POD 3 (SMD = −1.26 [−3.74 to 1.22]), POD 5 (SMD = −0.78 [−3.44 to 1.89]), POD 7 (SMD = 0.48 [−3.35 to 4.30]), and POD 10 (SMD = −0.41 [−1.19 to 0.37]) (Table 2).

Similarly, PRP administration did not reduce postoperative bleeding (Figure 3B). Primary postoperative bleeding was not estimable due to the absence of events in the included studies, and secondary bleeding showed no significant difference between the PRP and control groups (OR = 0.28 [0.07 to 1.18]; I^2^ = 0.0).

Substantial heterogeneity was observed across most pain-related analyses, likely reflecting methodological and population differences among studies, including variability in PRP preparation, pain-assessment methods, and evaluation timing. Overall, the current evidence does not support a consistent clinical benefit of intraoperative PRP in reducing postoperative pain or bleeding in children undergoing tonsillectomy.

### 3.3. Sensitivity Analysis

For adult postoperative pain, visual inspection of the funnel plot showed no clear asymmetry and Egger’s regression test was not statistically significant (*p* > 0.05).

Leave-one-out sensitivity analyses were performed to evaluate the influence of individual studies on the pooled estimates. In adults, the pooled effect on postoperative pain remained statistically significant and in the same direction after the sequential exclusion of any single trial, indicating that the adult finding was robust, although substantial heterogeneity persisted. In the pediatric analysis, by contrast, the pooled estimate was sensitive to individual studies. In particular, exclusion of the trial by Sidman et al. [16] resulted in a pooled estimate whose confidence interval no longer included the null value, which is consistent with the small number of pediatric trials and their substantial heterogeneity.

## 4. Discussion

The present meta-analysis identified different patterns of clinical benefit between adult and pediatric populations. PRP significantly reduced postoperative pain and improved wound healing in adults. In children, no significant reduction in postoperative pain was observed. The analgesic effect was most pronounced during the early postoperative period and gradually diminished over time. In contrast, PRP did not significantly reduce postoperative bleeding in either age group. Collectively, these findings indicate that intraoperative PRP may provide short term symptomatic relief and promote mucosal healing in adult patients undergoing tonsillectomy, whereas its clinical benefit in pediatric populations remains uncertain.

PRP is an autologous biologic preparation that contains a high concentration of platelets and releases multiple growth factors including platelet-derived growth factor, vascular endothelial growth factor, and transforming growth factor-β, which stimulate angiogenesis, enhance epithelial regeneration, and promote hemostasis [9,25]. In addition, studies using various wound models have shown that PRP contains cytokines and adhesive proteins such as fibrinogen and fibronectin, which support cell migration and modulate local inflammatory responses [7,26,27]. These biological properties provide a mechanistic rationale for the potential benefits of PRP in reducing postoperative pain and bleeding following tonsillectomy. A previous meta-analysis demonstrated that intraoperative PRP significantly reduced early postoperative pain and decreased the overall incidence of post-tonsillectomy hemorrhage [10]. However, the present age-stratified analysis identified that statistically significant benefits were observed primarily in adults, whereas pediatric evidence was limited to pain and bleeding outcomes and did not demonstrate a consistent benefit.

The observed age-related differences in PRP efficacy may reflect structural and immunological variations in tonsillar tissue. Histopathological analyses of adult tonsils with chronic tonsillitis have revealed a higher degree of interfollicular fibrosis and epithelial damage, which correlate with the severity of throat-related symptoms and tend to become more pronounced with increasing age [28,29]. Additionally, age-related alterations in the innate immune system of the tonsils have also been documented, potentially affecting local inflammatory responses and tissue repair dynamics [30,31]. Clinically, adults are known to experience greater and more prolonged postoperative pain compared with children, even under similar surgical and analgesic conditions [32]. Taken together, these structural and immunological differences may create a larger therapeutic substrate for PRP in adults. The more pronounced chronic inflammation, interfollicular fibrosis, and epithelial injury that accumulate with age provide more targets for the growth factors and anti-inflammatory cytokines released by activated platelets [6,7]. In children, whose tonsillar mucosa is less fibrotic and whose postoperative recovery is generally faster, the marginal gain from exogenous growth factors may be correspondingly smaller. One possible explanation is that the faster mucosal healing observed in children leaves less opportunity for PRP to provide additional therapeutic benefit.

In adults, PRP yielded a significant reduction in postoperative pain (SMD = −1.26 [95% CI −1.70 to −0.81]), particularly during the early recovery period (POD 0 to 5). The analgesic benefit gradually declined after the first postoperative week and was no longer statistically significant beyond POD 7. This time-dependent pattern suggests that PRP appears to exert its primary therapeutic effect during the acute inflammatory phase, when platelet-derived growth factors and bioactive mediators modulate the inflammatory response, promote epithelial regeneration, and reduce nociceptive stimulation. As tissue repair progresses and inflammation subsides, the additional benefit of PRP appears to diminish. Clinically, this early reduction in pain may improve patient comfort, facilitate earlier resumption of oral intake, and shorten the period of greatest postoperative discomfort.

However, substantial heterogeneity was observed in most pain analyses for both adults and children, with I^2^ values exceeding 90%. Several methodological factors are likely to have contributed to this variability. First, PRP preparation was not standardized across studies. Centrifugation protocols, platelet concentration, leukocyte content, and the method and timing of PRP activation varied or were incompletely reported, all of which are known to influence the biological activity of PRP [33,34]. Second, the volume and method of PRP application differed across studies and may have affected treatment efficacy. Third, both split-mouth and parallel-group study designs were included, and these designs differ in their ability to control for within-participant variability, which may have further contributed to the observed heterogeneity. Consequently, although most point estimates favored PRP, the magnitude of the analgesic effect varied considerably and should be interpreted with caution. In contrast, postoperative bleeding outcomes showed no statistical heterogeneity across studies (I^2^ = 0%), regardless of patient age. This finding suggests that procedural variations had less influence on postoperative bleeding than on pain outcomes. Although both pain and bleeding outcomes were derived from the same surgical settings within each study, the degree of heterogeneity markedly differed between them. This discrepancy is more likely explained by differences in PRP preparation and by patient-specific factors such as inflammatory response and pain sensitivity rather than by the surgical procedure itself. Pain perception inherently varies across individuals, whereas postoperative bleeding represents a more objective and uniform outcome.

The pooled analysis demonstrated a statistically significant improvement in wound healing (SMD = −1.43 [95% CI −2.65 to −0.22]) in adults. Although individual time points did not consistently reach statistical significance, the overall direction indicated accelerated epithelialization in the PRP group. This likely reflects a cumulative effect of PRP during the healing process rather than an isolated time-specific benefit. Differences in outcome grading systems and evaluation timing probably contributed to the substantial heterogeneity observed in the overall analysis. For example, Nanditha et al. [20] used a four-point epithelialization scale, whereas Wiriyaamornchai et al. [24] applied a five-stage morphological classification, which made direct comparisons across studies challenging. In pediatric tonsillectomy, Saad et al. [21] reported faster mucosal recovery on the PRP-treated side at POD 5 and 10, although the assessment relied solely on visual inspection without a standardized grading system. Overall, quantitative data on epithelialization after tonsillectomy remain limited, and future randomized controlled trials using objective and reproducible metrics are warranted to better define the regenerative role of PRP.

From a clinical perspective, the age-related findings of the present study have important implications for perioperative management. In adults, the analgesic benefit of PRP was concentrated within the first postoperative week, which corresponds to the period of greatest postoperative pain and functional impairment. During this interval, pain commonly interferes with oral intake, hydration, and sleep. Therefore, intraoperative PRP may serve as a useful adjunct to multimodal postoperative pain management by improving early postoperative recovery and facilitating earlier resumption of oral intake. Because PRP is an autologous biological product, it has a favorable safety profile with minimal immunological or infectious risk and can be prepared from a relatively small volume of the patient’s own blood. However, its use requires additional operative time and additional costs for specialized equipment and consumable materials. These practical considerations should be balanced against a clinical benefit that appears to be limited primarily to the early postoperative period. In contrast, no consistent improvement in postoperative pain or bleeding was observed in children. The pediatric pain findings were less stable than those observed in adults. The statistical significance of the pooled estimate changed following exclusion of one individual study, reflecting the small number of available pediatric trials and substantial between study heterogeneity. Overall, the present findings support consideration of PRP primarily in adult patients rather than routine application across all age groups. Unlike previous meta-analyses [10] that evaluated adults and children together, the present study demonstrated that the observed clinical effects of PRP differed between the adult and pediatric subgroup analyses. This age stratified approach provides a more clinically relevant framework for perioperative decision making and may help explain the inconsistent findings reported in previous pooled analyses.

This meta-analysis has several limitations. First, only nine trials were available, with three to five contributing to each pooled outcome, which limited statistical power and generalizability. Second, substantial methodological heterogeneity among the included studies may have influenced the pooled effect estimates despite the use of random effects models. Third, several trials used a split-mouth design in which the two tonsillar beds were treated as independent groups. Because the within-patient correlation was not incorporated, the precision of the corresponding pooled estimates may have been overestimated. Fourth, wound-healing outcomes were evaluated using non-standardized scales, impeding consistent quantitative synthesis. Fifth, only English language publications were included, which may have introduced language bias and limited the inclusion of potentially relevant studies published in other languages. Future large-scale, multicenter randomized controlled trials employing standardized PRP protocols and validated outcome measures are needed to confirm these findings and assess long-term safety and clinical efficacy.

## 5. Conclusions

Given the small number of included trials and substantial methodological heterogeneity, PRP may be considered a potential adjunct for reducing early postoperative morbidity in adults undergoing tonsillectomy. However, the current evidence is insufficient to support routine application, particularly in pediatric patients. Further well-designed multicenter randomized controlled trials using standardized PRP preparation protocols and outcome measures are required to confirm its efficacy and long-term safety.

## Figures and Tables

**Table 1:** Characteristics of included studies.

Study	Country	N	Age (Years)	Tonsillectomy Method	Comparator	PRP Preparation and Administration	Amount of Blood Collected	Duration of Follow-Up	Outcomes
Sidman (2008) [16]	USA	57	4–15	Not specified	Control (no PRP)	Autologous PRP prepared intraoperatively and applied as a gel membrane on both tonsillar beds.	30 mL	10 days	pain score
Salah Eldin (2011) [17]	Saudi Arabia	38	18–40	Dissection; haemostasis with bipolar diathermy	Contralateral side	Autologous PRP prepared by double centrifugation and applied as a gel membrane to one tonsillar bed.	25 mL	10 days	pain score postoperative bleeding
Gökçe Kutuk (2019) [18]	Turkey	80	4–16	Cold dissection; haemostasis with bipolar electrocautery	Tonsillectomy alone	Autologous PRP prepared by two-step centrifugation and applied as a gel membrane bilaterally.	25 mL	10 days	pain score postoperative bleeding
Chettri (2019) [19]	India	36	7–35	Dissection with ligation	Contralateral side	Autologous PRP prepared by double centrifugation, activated with calcium gluconate, and applied as a gel membrane on one side.	10 mL	36 h	pain score postoperative bleeding
Nanditha (2021) [20]	India	56	18–45	Not specified	Control (no PRP)	Autologous PRP prepared by two-step centrifugation and topically applied as a gel membrane to both fossae.	10 mL	14 days	pain score postoperative bleeding grading of tonsillar healing
Saad (2021) [21]	Egypt	40	4–15	Dissection and ligation	Contralateral side	Autologous PRP prepared by double centrifugation and applied as a gel membrane to one tonsillar bed.	25 mL	10 days	pain score postoperative bleeding grading of tonsillar healing
Rasheed (2023) [22]	Iraq	64	6–10	Dissection; haemostasis with ligation	Contralateral side	Autologous PRP prepared by double centrifugation and applied as a gel membrane to one tonsillar bed.	10 mL	10 days	postoperative bleeding
Muskaan (2025) [23]	India	41	18–40	Dissection and snare method	Contralateral fossa	Autologous PRP prepared by two-step centrifugation and applied as a gel membrane to one tonsillar fossa.	10 mL	10 days	pain score grading of tonsillar healing
Wiriyaamornchai (2025) [24]	Thailand	30	18–60	Extracapsular dissection using bipolar electrocautery	Contralateral side (saline injection)	Autologous PRP prepared using a commercial kit and injected (PRP injection) into one tonsillar bed.	20 mL	28 days	pain score postoperative bleeding grading of tonsillar healing

N indicates the number of participants included in the quantitative analyses.

**Table 2:** Subgroup analysis by postoperative day for pain and wound healing outcomes.

	Postoperative Pain in Adults	Postoperative Pain in Children	Wound Healing in Adults
Overall	SMD = −1.2557 [−1.7017; −0.8097] I^2^ = 92.2%	SMD = −0.7673 [−1.6871; 0.1525] I^2^ = 96.9%	SMD = −1.4346 [−2.6463; −0.2230] I^2^ = 94.6%
Operation day	N = 4 −1.0205 [−1.6665; −0.3744] I^2^ = 82.3%	N = 3 −1.3698 [−3.6637; 0.9241] I^2^ = 97.2%	
POD 1	N = 5 −1.4656 [−2.6882; −0.2429] I^2^ = 94.9%	N = 3 −0.8924 [−3.5738; 1.7891] I^2^ = 98.3%	N = 2 −0.9771 [−2.2637; 0.3095] I^2^ = 92.0%
POD 3	N = 3 −1.8947 [−3.1572; −0.6322] I^2^ = 92.0%	N = 2 −1.2613 [−3.7416; 1.2191] I^2^ = 97.5%	
POD 5	N = 3 −1.2698 [−2.5212; −0.0184] I^2^ = 94.3%	N = 3 −0.7775 [−3.4421; 1.8871] I^2^ = 98.2%	
POD 7	N = 3 −1.2819 [−2.6703; 0.1065] I^2^ = 94.8%	N = 2 0.4759 [−3.3513; 4.3031] I^2^ = 98.8%	N = 2 −1.4246 [−3.5035; 0.6544] I^2^ = 95.7%
POD 10	N = 2 −0.8676 [−2.5768; 0.8416] I^2^ = 96.0%	N = 2 −0.4081 [−1.1877; 0.3715] I^2^ = 80.3%	
POD 14	N = 1 0.0000 [−0.5061; 0.5061] I^2^ = NA		N = 2 −1.9571 [−5.8259; 1.9118] I^2^ = 98.2%
*p*-value	0.0223	0.9353	0.8595

POD, postoperative day; SMD, standardized mean difference. Values are presented as standardized mean differences (SMDs) with 95% confidence intervals (CIs). I^2^ represents the percentage of total variation across studies due to heterogeneity. N indicates the number of studies included in each subgroup. *p*-values indicate subgroup differences between time points.

## Data Availability

All data supporting the findings of this study were extracted from previously published articles cited in the manuscript. No new datasets were generated or analyzed during the current study. The extracted data are available from the corresponding author upon reasonable request.

## Supplementary Materials

The following supporting information can be downloaded at: https://www.mdpi.com/article/10.3390/jcm15166182/s1, Table S1: PRISMA 2020 checklist [35]; Table S2: Search strategy for each database; Table S3: Individual randomized controlled trial methodological quality.

## Abbreviations

The following abbreviations are used in this manuscript:

CI	Confidence Interval
I^2^	I-squared Statistic
OR	Odds Ratio
PICOS	Population, Intervention, Comparison, Outcomes, and Study Design
POD	Postoperative Day
PRISMA	Preferred Reporting Items for Systematic Reviews and Meta-Analyses
PRP	Platelet-Rich Plasma
RoB	Risk of Bias
SMD	Standardized Mean Difference
VAS	Visual Analogue Scale
