# Supplementary material for: Age-Specific Efficacy of Platelet-Rich Plasma in Tonsillectomy: A Systematic Review and Meta-Analysis
Source: J Clin Med. 2026 Aug 10;15(16):6182. doi: 10.3390/jcm15166182 (PMC13513369; doi:10.3390/jcm15166182)
Supplement: Supplementary file 1 [file jcm-15-06182-s001.zip › jcm-4424498-supplementary.pdf]

Supplementary Table S1. PRISMA 2020 checklist

Section and Topic	Item #	Checklist item	Location where item is reported
**TITLE**			
Title	1	Identify the report as a systematic review.	Title, Lines 2–3
**ABSTRACT**			
Abstract	2	See the PRISMA 2020 for Abstracts checklist.	Abstract, Lines 12–31
**INTRODUCTION**			
Rationale	3	Describe the rationale for the review in the context of existing knowledge.	Section 1 (Introduction), Lines 36–73
Objectives	4	Provide an explicit statement of the objective(s) or question(s) the review addresses.	Section 1 (Introduction), Lines 74–80
**METHODS**			
Eligibility criteria	5	Specify the inclusion and exclusion criteria for the review and how studies were grouped for the syntheses.	Section 2.1, Lines 89–93 (PICOS) and Lines 106–113
Information sources	6	Specify all databases, registers, websites, organisations, reference lists and other sources searched or consulted to identify studies. Specify the date when each source was last searched or consulted.	Section 2.1, Lines 93–101 (PubMed, Embase, Scopus, Google Scholar, Cochrane CENTRAL; reference lists; last searched October 2025)
Search strategy	7	Present the full search strategies for all databases, registers and websites, including any filters and limits used.	Section 2.1, Lines 97–98; full strategies in Supplementary Table S1
Selection process	8	Specify the methods used to decide whether a study met the inclusion criteria of the review, including how many reviewers screened each record and each report retrieved, whether they worked independently, and if applicable, details of automation tools used in the process.	Section 2.1, Lines 99–105; Figure 1
Data collection process	9	Specify the methods used to collect data from reports, including how many reviewers collected data from each report, whether they worked independently, any processes for obtaining or confirming data from study investigators, and if applicable, details of automation tools used in the process.	Section 2.2, Lines 117–135
Data items	10a	List and define all outcomes for which data were sought. Specify whether all results that were compatible with each outcome domain in each study were sought (e.g. for all measures, time points, analyses), and if not, the methods used to decide which results to collect.	Section 2.2, Lines 120–129
	10b	List and define all other variables for which data were sought (e.g. participant and intervention characteristics, funding sources). Describe any assumptions made about any missing or unclear information.	Section 2.2, Table 1
Study risk of bias assessment	11	Specify the methods used to assess risk of bias in the included studies, including details of the tool(s) used, how many reviewers assessed each study and whether they worked independently, and if applicable, details	Section 2.2, Lines 136–141; Supplementary Table S2

Section and Topic	Item #	Checklist item	Location where item is reported
		of automation tools used in the process.	
Effect measures	12	Specify for each outcome the effect measure(s) (e.g. risk ratio, mean difference) used in the synthesis or presentation of results.	Section 2.3, Lines 143–151
Synthesis methods	13a	Describe the processes used to decide which studies were eligible for each synthesis (e.g. tabulating the study intervention characteristics and comparing against the planned groups for each synthesis (item #5)).	Section 2.1, Lines 106–113
	13b	Describe any methods required to prepare the data for presentation or synthesis, such as handling of missing summary statistics, or data conversions.	Section 2.3, Lines 148–151
	13c	Describe any methods used to tabulate or visually display results of individual studies and syntheses.	Table 1, Table 2, Figures 2–3
	13d	Describe any methods used to synthesize results and provide a rationale for the choice(s). If meta-analysis was performed, describe the model(s), method(s) to identify the presence and extent of statistical heterogeneity, and software package(s) used.	Section 2.3, Lines 152-157
	13e	Describe any methods used to explore possible causes of heterogeneity among study results (e.g. subgroup analysis, meta-regression).	Section 2.3, Lines 1572-157, Sections 3.1–3.2 and Table 2
	13f	Describe any sensitivity analyses conducted to assess robustness of the synthesized results.	Section 2.3, Lines 161–162
Reporting bias assessment	14	Describe any methods used to assess risk of bias due to missing results in a synthesis (arising from reporting biases).	Section 2.3, Lines 158–161
Certainty assessment	15	Describe any methods used to assess certainty (or confidence) in the body of evidence for an outcome.	Not reported.
**RESULTS**			
Study selection	16a	Describe the results of the search and selection process, from the number of records identified in the search to the number of studies included in the review, ideally using a flow diagram.	Figure 1
	16b	Cite studies that might appear to meet the inclusion criteria, but which were excluded, and explain why they were excluded.	Figure 1; exclusion criteria in Section 2.1, Lines 110–113
Study characteristics	17	Cite each included study and present its characteristics.	Section 3; Table 1
Risk of bias in studies	18	Present assessments of risk of bias for each included study.	Supplementary Table S2
Results of individual studies	19	For all outcomes, present, for each study: (a) summary statistics for each group (where appropriate) and (b) an effect estimate and its precision (e.g. confidence/credible interval), ideally using structured tables or plots.	Figures 2–3; Table 2
Results of syntheses	20a	For each synthesis, briefly summarise the characteristics and risk of bias among contributing studies.	Section 3.1, Lines 171–175; Section 3.2, Lines 207–208 and 227–231; Table 1;

Section and Topic	Item #	Checklist item	Location where item is reported
			Supplementary Table S2
	20b	Present results of all statistical syntheses conducted. If meta-analysis was done, present for each the summary estimate and its precision (e.g. confidence/credible interval) and measures of statistical heterogeneity. If comparing groups, describe the direction of the effect.	Section 3.1, Lines 183–191 and 197–204; Section 3.2, Lines 216–226; Table 2; Figures 2–3
	20c	Present results of all investigations of possible causes of heterogeneity among study results.	Section 3.1, Lines 185–191; Section 3.2, Lines 216–222; Table 2
	20d	Present results of all sensitivity analyses conducted to assess the robustness of the synthesized results.	Section 3.3
Reporting biases	21	Present assessments of risk of bias due to missing results (arising from reporting biases) for each synthesis assessed.	Section 3.3
Certainty of evidence	22	Present assessments of certainty (or confidence) in the body of evidence for each outcome assessed.	Not reported.
**DISCUSSION**			
Discussion	23a	Provide a general interpretation of the results in the context of other evidence.	Section 4, Lines 244–252
	23b	Discuss any limitations of the evidence included in the review.	Section 4, Lines 294–313 and 351–361
	23c	Discuss any limitations of the review processes used.	Section 4, Lines 359–361
	23d	Discuss implications of the results for practice, policy, and future research.	Section 4, Lines 361–364; Section 5
**OTHER INFORMATION**			
Registration and protocol	24a	Provide registration information for the review, including register name and registration number, or state that the review was not registered.	Not registered.
	24b	Indicate where the review protocol can be accessed, or state that a protocol was not prepared.	A protocol was prepared a priori using the PICOS framework (Section 2, Lines 89–93) but was not published or made publicly accessible.
	24c	Describe and explain any amendments to information provided at registration or in the protocol.	Not applicable
Support	25	Describe sources of financial or non-financial support for the review, and the role of the funders or sponsors in the review.	Funding statement, Lines 379–380
Competing interests	26	Declare any competing interests of review authors.	Conflicts of Interest statement, Line 389

Section and Topic	Item #	Checklist item	Location where item is reported
Availability of data, code and other materials	27	Report which of the following are publicly available and where they can be found: template data collection forms; data extracted from included studies; data used for all analyses; analytic code; any other materials used in the review.	Data Availability Statement, Lines 385-388; Supplementary Tables S1–S2. Analytic code is not publicly available.

From: Page MJ, McKenzie JE, Bossuyt PM, Boutron I, Hoffmann TC, Mulrow CD, et al. The PRISMA 2020 statement: an updated guideline for reporting systematic reviews. BMJ 2021;372:n71. doi: 10.1136/bmj.n71

For more information, visit: http://www.prisma-statement.org/

Supplementary Table S2. Search strategy for each database

Database / Source	Search terms / query	Filters / limits applied
PubMed (MEDLINE)	(("platelet-rich plasma"[All Fields] OR "PRP"[All Fields]) AND (tonsillectomy[MeSH Terms] OR tonsillectomy[Title/Abstract] OR adenotonsillectomy[Title/Abstract]) AND (pain[Title/Abstract] OR bleeding[Title/Abstract] OR hemorrhage[Title/Abstract] OR haemorrhage[Title/Abstract] OR healing[Title/Abstract] OR epithelialization[Title/Abstract] OR "tonsillar fossa"[Title/Abstract]))	Humans; English; no date limit; no study-design filter
Embase	('platelet rich plasma'/exp OR 'platelet-rich plasma':ab,ti OR PRP:ab,ti) AND ('tonsillectomy'/exp OR tonsillectomy:ab,ti OR adenotonsillectomy:ab,ti) AND (pain:ab,ti OR bleeding:ab,ti OR hemorrhage:ab,ti OR haemorrhage:ab,ti OR healing:ab,ti OR epithelialization:ab,ti OR 'tonsillar fossa':ab,ti)	Humans; English; no trial filter; no date limit
Scopus	TITLE-ABS-KEY ("platelet-rich plasma" OR PRP) AND TITLE-ABS-KEY (tonsillectomy OR adenotonsillectomy) AND TITLE-ABS-KEY (pain OR bleeding OR hemorrhage OR haemorrhage OR healing OR epithelialization OR "tonsillar fossa")	Document type: Article; Language: English; no date limit
Cochrane CENTRAL	("platelet-rich plasma" OR PRP) AND (tonsillectomy OR adenotonsillectomy)	Trials only (database default); no date limit; Language not restricted at search
Google Scholar	"platelet-rich plasma" AND tonsillectomy AND (pain OR bleeding OR hemorrhage OR haemorrhage OR healing OR epithelialization OR "tonsillar fossa")	English; first 200 results screened; backward and forward citation tracking performed

Supplemental Table S3. Individual randomized controlled trial methodological quality.

Study	Random sequence generation	Allocation concealmen t	Blinding of participants and personnel	Blinding of outcome assessment	Incomplete outcome data addressed	Free of selective reporting	Risk of Bias of randomized studies
Sidman 2008	Yes	Yes	Yes	No	No	No	high
Salah Eldin 2011	Yes	Yes	Yes	Yes	Yes	Yes	low
Gokce Kutuk 2019	Yes	Yes	Yes	Yes	Yes	Yes	low
Chettri 2019	Yes	Yes	Yes	Yes	Yes	Yes	low
Nanditha 2021	Yes	Yes	Yes	Yes	Yes	Yes	low
Saad 2021	Unclear	Yes	Yes	Yes	Yes	Unclear	Unclear
Rasheed 2023	Yes	Yes	Yes	No	Unclear	Yes	high
Muskaan 2025	Yes	Yes	Yes	No	Unclear	Yes	high
Wiriyaamor nchai 2025	Yes	Yes	Yes	Yes	Yes	Yes	low
